# Mercury-associated minimal change disease induced by whitening cream: a case report

**DOI:** 10.3389/fmed.2026.1931047

**Published:** 2026-08-19

**Authors:** Wen-Jing Zhou, Xiao-Na Li, Wen Zhang, Yu-Qiong Yang

**Affiliations:** Department of Nephrology, The Third People's Hospital of Yunnan Province, Kunming, Yunnan, China

**Keywords:** chelation therapy, mercury poisoning, minimal change disease, nephrotic syndrome, skin-whitening cosmetics

## Abstract

Mercury-associated minimal change disease (M-MCD) is a rare form of secondary minimal change disease (MCD) caused by chronic mercury exposure. Given its rarity and nonspecific clinical presentation, M-MCD is frequently underrecognized in clinical practice. We report the case of a 32-year-old woman who presented with nephrotic syndrome following 4 months of applying a skin-whitening cream. Markedly elevated urinary mercury concentrations were detected, and renal biopsy confirmed MCD with immunoglobulin M (IgM) deposition. The patient received four courses of sodium dimercaptopropane sulfonate chelation therapy combined with glucocorticoids. Complete remission of proteinuria was achieved within 1 month, accompanied by normalization of urinary mercury levels. This case underscores the necessity of obtaining a detailed exposure history in patients with unexplained nephrotic syndrome, particularly among young women using skin-whitening cosmetics. Prompt recognition of mercury exposure and timely chelation therapy are essential for achieving remission; adjunctive glucocorticoids may be considered in selected patients with severe disease or suspected immune- mediated injury.

## 1 Introduction

Mercury is a well-established nephrotoxic heavy metal typically acquired through occupational or environmental exposure. Increasingly, the prolonged application of cosmetics containing unregulated mercury levels has emerged as a non-negligible source of exposure (1, 2). The most common renal manifestation of chronic mercury poisoning is nephrotic syndrome, with membranous nephropathy and minimal change disease (MCD) representing the predominant histopathological patterns (3, 4). Mercury-associated minimal change disease (M-MCD) is clinically and histopathologically indistinguishable from primary minimal change disease (P-MCD), making accurate diagnosis challenging and increasing the risk of misdiagnosis. Herein, we present the case of a 32-year-old woman who developed M-MCD following the use of a skin-whitening cream and review the relevant literature on its clinicopathological characteristics, diagnostic approaches, and treatment strategies to improve clinical recognition and optimize patient management.

## 2 Case presentation

A 32-year-old woman presented with bilateral lower-extremity edema that had persisted for more than 1 month. She first noticed marked bilateral pitting edema accompanied by foamy urine and decreased urine output (approximately 800–1,000 mL/d) 1 month before admission. She denied gross hematuria, chest tightness, dyspnea, oral ulcers, arthralgia, or other systemic symptoms. Her past medical history was unremarkable. On physical examination, marked bilateral pitting edema of the lower extremities was noted. The facial skin tone was visibly lighter than that of the rest of the body, without evidence of desquamation, erythema, atrophy, or pigmentation abnormalities. No gingival mercury lines or other significant physical abnormalities were identified. Laboratory investigations demonstrated nephrotic-range proteinuria, with urinalysis showing proteinuria (3+) and a 24-h urinary protein excretion of 8.64 g/24 h. Blood biochemical analysis revealed total protein 46.0 g/L, albumin 17.1 g/L, aspartate aminotransferase 13.6 U/L, alanine aminotransferase 20.1 U/L, total cholesterol 11.19 mmol/L, triglycerides 2.34 mmol/L, high-density lipoprotein cholesterol 2.76 mmol/L, low-density lipoprotein cholesterol 6.73 mmol/L, and serum creatinine 86 μmol/L. Immunological testing revealed an immunoglobulin G level of 3.59 g/L, an immunoglobulin M level of 4.04 g/L, and a complement C4 level of 0.48 g/L. The erythrocyte sedimentation rate was elevated at 51 mm/h. Results of the complete blood count, anti-neutrophil cytoplasmic antibody testing, antinuclear antibody panel, serum and urine protein electrophoresis, serum and urine immunofixation electrophoresis, serum and urine free light-chain assays, and virological investigations were unremarkable. Electrocardiography, chest computed tomography, and urinary tract ultrasonography showed no significant abnormalities. Detailed laboratory findings are summarized in Table 1.

**Table 1:** The specific laboratory test results of this case.

Item	Result	Reference range
Complete blood count		
White blood cell count (/L)	6.75 × 10^9^	3.5–9.5 × 10^9^
Red blood cell count (/L)	5.63 × 10^9^	3.8–5.1 × 10^9^
Hemoglobin (g/L)	145	115–150
Platelet count (/L)	296 × 10^9^	125–350 × 10^9^
Urinalysis		
Urine protein	3+	Negative
Urinary red blood cells (/HPF)	2–3	0–2
24-h urine protein (g/24 h)	8.64	0–0.14
Blood biochemistry		
Total protein (g/L)	46.0	60–80
Albumin (g/L)	17.1	35–52
Aspartate aminotransferase (U/L)	13.6	0–32
Alanine aminotransferase (U/L)	20.1	0–33
Total cholesterol (mmol/L)	11.19	2.8–5.7
Triglycerides (mmol/L)	2.34	0.23–1.71
HDL cholesterol (mmol/L)	2.76	1.04–1.42
LDL cholesterol (mmol/L)	6.73	<2.07
Serum creatinine (μmol/L)	86	35–100
Immunoglobulin G (IgG) (g/L)	3.59	7–16
Immunoglobulin A (IgA) (g/L)	2.17	0.7–4
Immunoglobulin M (IgM) (g/L)	4.04	0.4–2.3
Complement C3 (g/L)	1.11	0.9–1.8
Complement C4 (g/L)	0.48	0.13–0.43
Erythrocyte sedimentation rate (mm/h)	51	0–20
Antinuclear antibody profile	Negative	Negative
Anti-neutrophil cytoplasmic antibody	Negative	Negative
HBV, HCV, HIV, Syphilis	Negative	Negative
Serum immunofixation electrophoresis	No monoclonal globulins	
Urine immunofixation electrophoresis	No monoclonal globulins	
Serum protein electrophoresis	No M protein detected	
Urine protein electrophoresis	No M protein detected	
Serum free kappa light chain (mg/L)	24.25	3.30–19.40
Serum free lambda light chain (mg/L)	28.77	5.71–26.30
Serum free kappa/lambda ratio	0.84	0.26–1.65
Urine free kappa light chain (mg/L)	315.4	1.17–86.46
Urine free lambda light chain (mg/L)	205.54	0.27–15.21
Urine free kappa/lambda ratio	1.53	1.83–14.26
Urinary mercury (μg/L)	82.63	0–10

**Figure 1:**
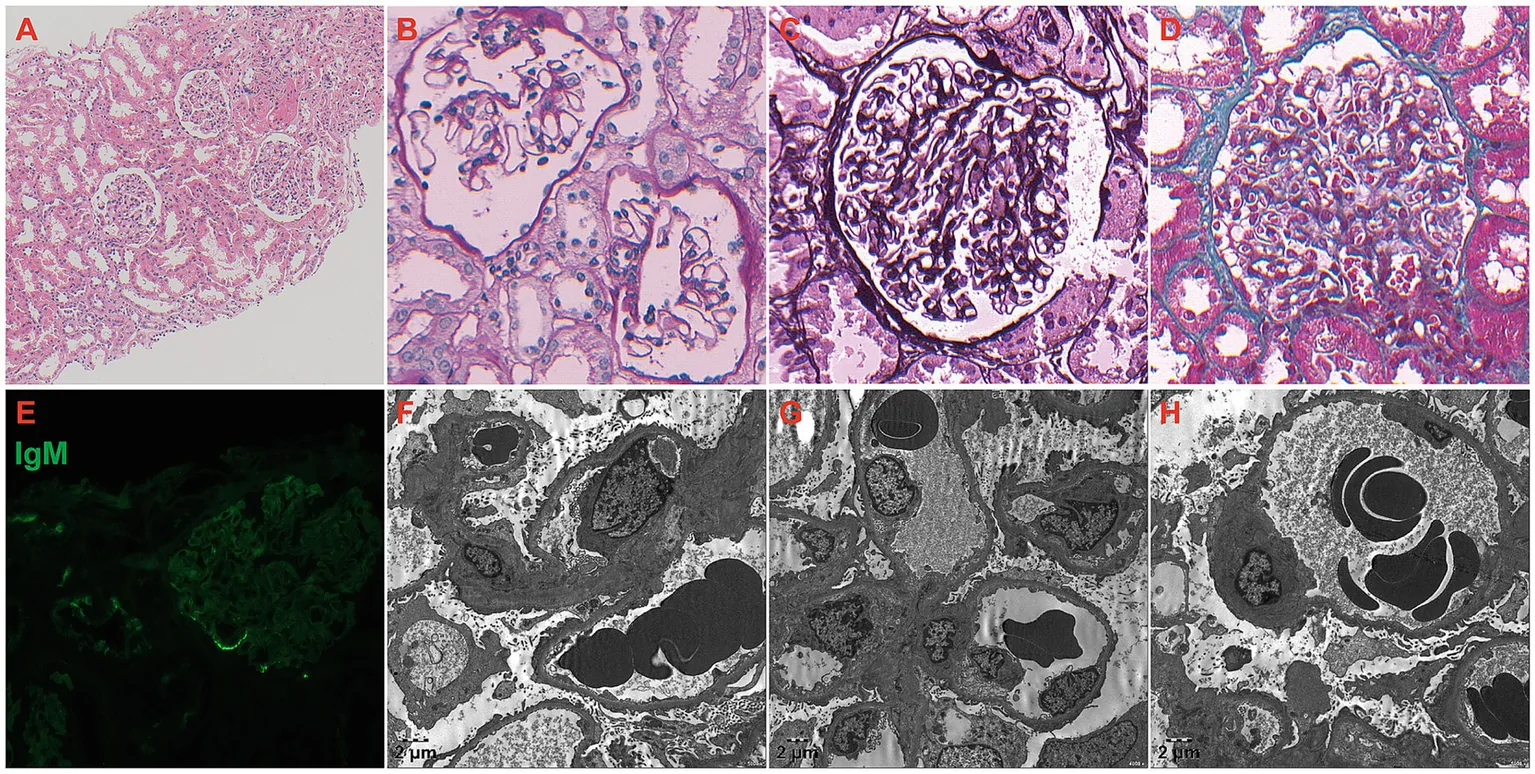
Pathological manifestations of the renal biopsy. **(A**–**D)** Light microscopy showed the glomerulus was generally normal; **(A)** HE staining (×100), **(B)** PAS staining (×400), **(C)** PASM staining (×400), **(D)** Masson staining (×400). **(E)** Immunofluorescence staining revealed deposits of IgM (+). **(F**–**H)** Electron microscopy indicated that podocytes were swollen with vacuolation and diffuse effacement of podocyte foot processes.

A renal biopsy was subsequently performed. Light microscopy identified 29 glomeruli, including 3 globally sclerotic glomeruli. The remaining glomeruli showed no significant mesangial hypercellularity or mesangial matrix expansion. No eosinophilic proteinaceous deposits were identified within the mesangial regions. The capillary loops were patent, and the glomerular basement membranes showed no significant thickening, spike formation, mesangial interposition, or double-contour formation. No subepithelial or subendothelial deposits were observed. No proliferation of parietal epithelial cells or crescent formation was observed. Tubular epithelial cells exhibited granular and vacuolar degeneration, with focal tubular dilatation. Focal loss of the brush border and focal tubular atrophy were noted. The renal interstitium demonstrated scattered lymphocytic infiltration without significant fibrosis. Small arteries showed no significant wall thickening, hyalinosis, or fibrinoid necrosis (Figures 1A–D). Immunofluorescence staining showed IgG (−), IgA (+/−), IgM (+), C3 (−), C1q (−), fibrinogen (−), and albumin (−), without significant immune complex deposition (Figure 1E). Electron microscopy demonstrated that podocytes were swollen with vacuolation, and diffuse effacement of podocyte foot processes was observed, with microvillus transformation involving more than 95% of the glomerular capillary surface. Segmental thickening of the glomerular basement membrane and scant mesangial electron-dense deposits were also observed (Figures 1F–H). Taken together, these pathological findings were most consistent with MCD, although early focal segmental glomerulosclerosis (FSGS) could not be completely excluded.

During further history-taking, the patient disclosed applying a skin-whitening cream twice daily for approximately 4 months before symptom onset. The product lacked identifiable manufacturer, production, and expiration details, and immediate discontinuation was advised. Urinary mercury testing revealed a markedly elevated mercury concentration of 82.63 μg/L (reference range, 0–10 μg/L). Combined with the clinical and pathological findings, this finding supported the diagnosis of M-MCD.

**Figure 2:**
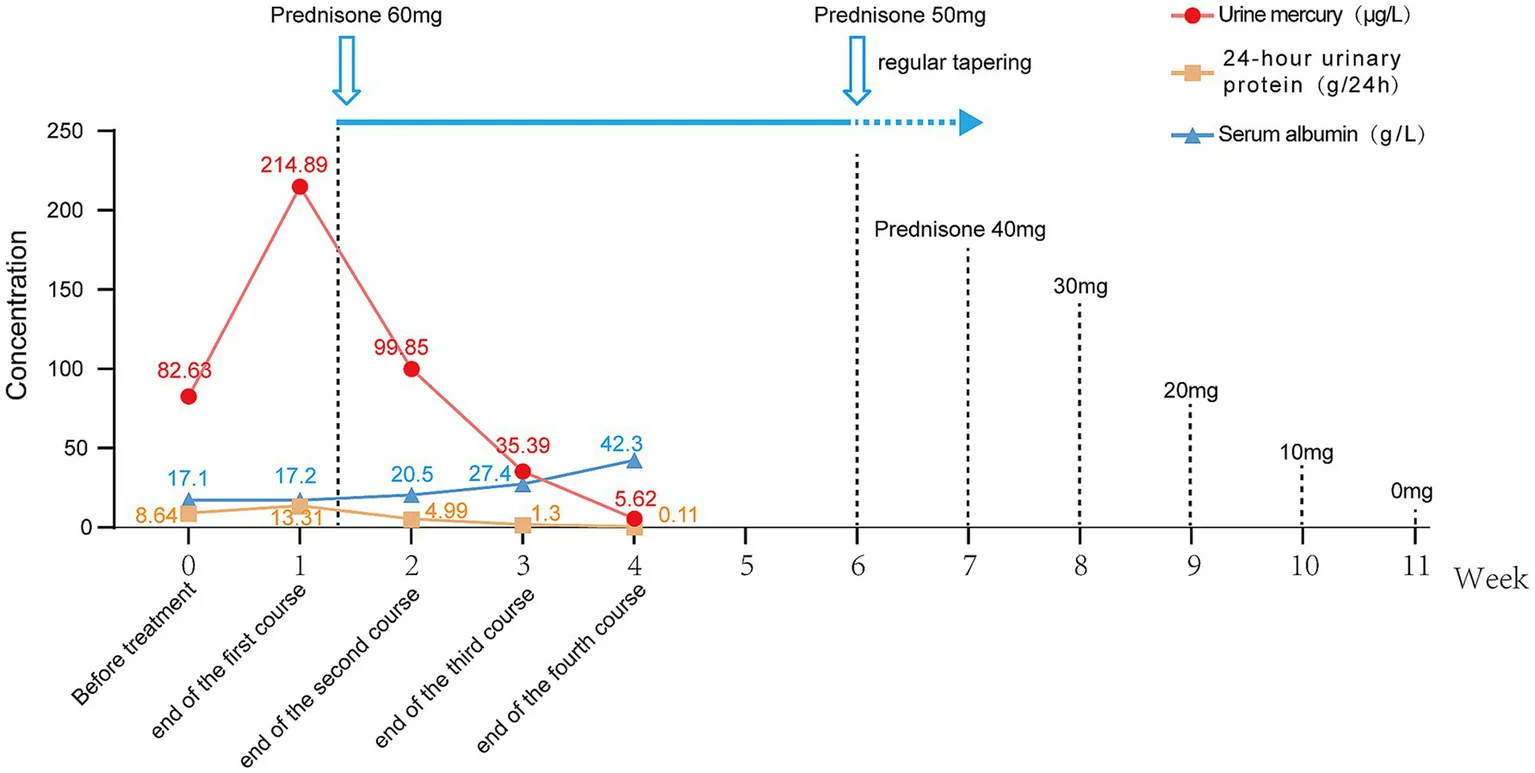
Clinical course and response to the treatments.

Chelation therapy with intramuscular sodium dimercaptopropane sulfonate (DMPS) was initiated at 250 mg/day. Each course comprised three consecutive days of administration followed by a 4-day interval, totaling four courses. After the first course, 24-h urinary protein excretion increased transiently to 13.31 g/24 h. Given the persistence of nephrotic-range proteinuria despite this early intervention, oral prednisone (60 mg/day) was added. Subsequently, urinary mercury and proteinuria steadily declined concurrently with a progressive recovery in serum albumin. The temporal dynamics of urinary mercury levels, proteinuria, and serum albumin are illustrated in Figure 2.

Following completion of the fourth course of DMPS therapy, urinalysis was negative for protein, urinary mercury had decreased to 5.62 μg/L, and serum albumin normalized to 42.3 g/L. Two weeks later, the prednisone dose was tapered by 10 mg weekly until complete cessation, with a total glucocorticoid treatment duration of 11 weeks. Concurrent supportive care comprised an angiotensin II receptor blocker, antioxidants, anticoagulants, diuretics, calcium supplementation, gastric mucosal protectants, and lipid-lowering agents. Over the follow-up period, the patient maintained stable renal function and sustained remission, with urinary mercury remaining within normal limits and no recurrence of proteinuria.

## 3 Discussion

Mercury is a widely distributed toxicant that can enter the human body through multiple occupational, environmental, and lifestyle-related exposure pathways. Historically, occupational exposure constituted the primary source of exposure, particularly among individuals employed in mercury mining, smelting, instrument manufacturing, and related industries (5). In recent years, however, increasing attention has been directed toward non-occupational mercury exposure. Notably, the prolonged application of mercury-adulterated skin-whitening cosmetics has emerged as a major cause of chronic toxicity, often characterized by an insidious onset and delayed diagnosis (1, 2). Other non-occupational sources include mercury-containing traditional medicines (2, 6) and the consumption of mercury-contaminated fish (7). The demographic profile strongly correlates with the exposure route: occupational mercury poisoning predominantly affects men aged 20–50 years, cosmetic-related mercury poisoning mainly occurs in young and middle-aged women, whereas mercury poisoning caused by traditional mercury-containing medicines shows no apparent age or sex predilection (4). The present case involved a 32-year-old woman who developed nephrotic syndrome following 4 months of applying an unregulated skin-whitening cream. Although the mercury content of the cosmetic product was not directly quantified, the patient’s markedly elevated urinary mercury level, long history of using the cosmetic, absence of other identifiable sources of mercury exposure, alongside clinical improvement following product cessation and chelation therapy, firmly implicated the cosmetic mercury exposure as the primary etiological trigger.

Mercury poisoning is a multisystem disorder with diverse and heterogeneous clinical manifestations. Given their central role in mercury accumulation and excretion, the kidneys serve as the primary targets of this heavy metal. A retrospective study reported renal involvement in 26.74% of patients with mercury poisoning, 89.13% of whom presented with nephrotic syndrome (3). In addition to renal involvement, mercury poisoning may affect the nervous system, skin, gastrointestinal system, hematologic system, and cardiovascular system, manifesting as tremor, peripheral neuropathy, insomnia, memory impairment (7–9), skin rash (10), abdominal pain, stomatitis, liver injury (1, 11, 12), thrombocytopenia (13), and hypertension (14). Nevertheless, such signs are largely nonspecific and highly variable; their complete absence in certain patients further exacerbates the risk of misdiagnosis. In the present case, the patient presented only with massive proteinuria, hypoalbuminemia, bilateral lower-extremity edema, and an unusually fair facial skin tone. Without the characteristic neurological, dermatological, or gastrointestinal manifestations of mercury poisoning, no suggestive physical signs such as a gingival mercury line were observed. Consequently, her clinical profile was indistinguishable from primary nephrotic syndrome. Relying solely on symptomatic presentation would have readily invited an erroneous diagnosis of P-MCD, thereby obscuring the critical history of mercury exposure.

Nephrotic syndrome is a common manifestation of mercury poisoning–associated renal disease, with membranous nephropathy and MCD representing the predominant pathological subtypes. Two studies evaluating renal biopsy findings in patients with mercury-associated renal disease reported that approximately 51% of cases were diagnosed as membranous nephropathy, 35–37% as MCD, and a minority as mesangial proliferative glomerulonephritis or FSGS (3, 4). In the present case, light microscopy revealed no significant glomerular proliferation or classic features of membranous nephropathy, whereas electron microscopy demonstrated diffuse podocyte foot process effacement, consistent with the pathological characteristics of MCD. Concurrently, immunofluorescence demonstrated positivity for IgM, with no definitive immune complex deposition identified. While immunofluorescence is typically negative in M-MCD, sporadic case reports have documented aberrant IgA or IgM deposition (15, 16), implying that mercury exposure may disrupt immune homeostasis and incite secondary renal immunoglobulin deposition. Notably, differentiation from early-stage FSGS is required during the pathological diagnostic process. Although focal segmental thickening of the glomerular basement membrane was noted in certain glomeruli, light microscopy revealed no definitive segmental sclerotic lesions. When integrated with the electron microscopy findings of diffuse foot process effacement and the rapid post-treatment resolution of proteinuria, the clinical picture strongly favors MCD over FSGS. Furthermore, although M-MCD and P-MCD share overlapping clinical and routine histopathological profiles, subtle ultrastructural variations exist. Compared with P-MCD, patients with M-MCD exhibit a significantly narrower mean podocyte foot process width (935.0 nm vs. 1403.2 nm), suggesting that their podocyte injury tends to be focal and heterogeneous rather than the diffuse, uniform injury seen in P-MCD (17). Although foot process morphometry is not yet a routine diagnostic criterion, a multimodal approach integrating exposure history, urinary toxicological screening, and comprehensive renal biopsy evaluation will markedly enhance the differentiation of M-MCD from P-MCD.

The pathogenic mechanisms driving M-MCD remain to be fully elucidated, though current evidence points to a dual etiology involving both direct cytotoxicity and immune-mediated pathways. Mercury introduces direct cytotoxic insult to podocytes due to the high affinity of Hg^2+^ for sulfhydryl (–SH) groups; its irreversible binding to intracellular thiol-containing proteins disrupts both structural integrity and cellular function (18). Mechanistically, mercury suppresses intracellular antioxidant enzymes, inciting an excessive accumulation of reactive oxygen species and subsequent oxidative stress (19). Additionally, mercury destabilizes the podocyte cytoskeletal network, resulting in foot process effacement and injury to the glomerular filtration barrier (20), which constitutes a key pathological basis for proteinuria in MCD. Parallel to direct toxicity, mercury acts as an orchestrator of immune dysregulation. Mercury can act as a hapten and bind to endogenous proteins to form new antigens, thereby activating CD4+ T lymphocytes, which release cytokines such as interleukin-13 and interleukin-17 (9, 21). These cytokines directly or indirectly impair podocyte function (22). Mercury exposure also stimulates autoantibody production; these circulating autoantibodies target podocytes, inducing structural and functional abnormalities that culminate in proteinuria (21, 23). Beyond these pathways, emerging data indicate that mercury may interfere with epigenetic regulatory mechanisms, including DNA methylation and histone modification, thereby modulating immune-related gene expression (24). This mercury-induced epigenetic reprogramming may explain protracted clinical courses post-chelation, which frequently necessitate adjunctive corticosteroid therapy to achieve full remission. In the current case, renal histopathology revealed diffuse foot process effacement and mild IgM deposition. Alongside a markedly elevated serum IgM concentration (4.04 g/L), these findings clinically substantiate the aforementioned dual pathogenic mechanisms of mercury toxicity.

Distinct from P-MCD, the therapeutic management of M-MCD centers on targeted heavy metal clearance rather than indiscriminate prolonged high-dose corticosteroid administration (3, 17). Once mercury exposure has been identified, all potential sources of exposure should be eliminated immediately. Standard chelation utilizes DMPS at 0.125–0.25 g/day, administered for three consecutive days followed by a 4-day interval (3). Guided by serial urinary mercury surveillance, patients typically require one to four courses. Accumulating evidence indicates that toxin avoidance coupled with standardized chelation suffices for most patients; A cohort study demonstrated that 77.8% of patients achieved complete remission via chelation monotherapy alone (17). Furthermore, comparative data revealed no statistically significant variance in complete remission rates among patients receiving chelation alone, chelation with glucocorticoids, or chelation combined with dual immunosuppression (3). Conversely, long-term and high-dose corticosteroid regimens carry substantial risks of systemic toxicities (25). Glucocorticoids are therefore excluded from routine protocols, reserved primarily for cases characterized by inadequate chelation response, rapid clinical deterioration, or pronounced immune activation (3). In our patient, 24-h urinary protein excretion increased transiently to 13.31 g/24 h following the first chelation course, consistently exceeding 10 g/24 h across repeated assays and accompanied by severe hypoalbuminemia (17.2 g/L). The precise cause of this fluctuation remains unclear, as multiple factors including fluid status, blood pressure, and disease progression influence urinary protein excretion. Notably, the patient exhibited clear evidence of immune activation, including glomerular IgM deposition and markedly elevated serum IgM (4.04 g/L). Given the persistent nephrotic-range proteinuria despite chelation therapy and the presence of these immune-activation markers, full-dose oral prednisone was initiated, followed by gradual tapering after proteinuria became negative, with a total glucocorticoid treatment duration of 11 weeks. Ultimately, the patient achieved complete clinical remission within 1 month of treatment initiation. This case highlights the importance of individualized treatment adjustment based on serial monitoring of urinary mercury, proteinuria, and clinical response. Table 2 summarizes the clinicopathological features and treatment strategies of reported cases of M-MCD. Moreover, M-MCD carries an excellent prognosis, exhibiting a remarkably lower relapse rate compared with P-MCD (0% vs. 47.1%) (17).

**Table 2:** Clinicopathological characteristics, treatment and prognosis of M-MCD.

Cases	Age/sex (number of cases)	Duration of mercury exposure (months)	Type of pathology (number of cases)	Urinary mercury (reference value)	24-h Urine Protein (g/24 h)	Treatment	Outcome	Time to complete remission (months)
Qin et al. (17)	21-50/F (19) 25-50/M (2)	The average was 4 months	MCD	13.6–130.1 μg/L (<10 μg/L)	5.8 ± 4.5	Seven patients received chelation therapy with DMPS; Seven patients received chelation therapy with DMPS+ short-term glucocorticoid; Two patients received chelation therapy with DMPS+ full-dose prednisone	CR	The median time was 2 months.
Tang et al. (26)	26-45/F (4)	2–6	MCD (3) MCD + IgA nephropathy (1)	316–2,521 nmol/d (< 35 nmol/d)	8.35–20.69	Two patients received chelation therapy with D-penicillamine. Two patients received chelation therapy with D-penicillamine+ glucocorticoid.	CR	1–9
Niu et al. (27)	39/F	6	MCD with IgA nephropathy	90 μg/g creatinine (<4 μg/g creatinine)	4.10	Chelation therapy with DMPS+ short-term glucocorticoid	CR	1
Gao et al. (15)	65/F	6	MCD with IgA deposition	27.5 μg/g creatinine (<4 μg/g creatinine)	3.54	Chelation therapy with DMPS	CR	0.75
Zhang et al. (28)	28/F	11	MCD	469 μmol/L (<50 μmol/L)	8.25	Chelation therapy with DMPS+ full-dose prednisone	CR	6
WĄGROWSKA-DANILEWICZ et al. (29)	42/M	8	MCD	830ug/L (<10 μg/L)	30	Chelation therapy with DMPS +glucocorticoid	CR	12
Tang et al. (30)	34/F	4	MCD	287 nmol/L (<50 nmol/L)	8.35	chelation therapy with D-penicillamine	CR	9
Zhang et al. (31)	40/F	6	MCD with IgA deposition	31.7 μg/g creatinine (<4 μg/g creatinine)	5	Chelation therapy with DMPS+ short-term glucocorticoid	CR	Not available
Dong et al. (16)	33/F	7	MCD with IgM deposition	96.6 μg/L (<10 μg/L)	5.44	Chelation therapy with DMPS +glucocorticoid	CR	1
This study	32/F	4	MCD with IgM deposition	82.63 μg/L (<10 μg/L)	8.64	Chelation therapy with DMPS +glucocorticoid	CR	1

F, female; M, male; MCD, minimal change disease; DMPS, dimercaptopropane sulfonate; CR, complete remission.

M-MCD represents a treacherous clinical masquerader that can flawlessly mimic P-MCD, frequently leading to delayed or erroneous diagnosis. Consequently, maintaining a high index of suspicion for toxicological etiologies is imperative when evaluating unexplained nephrotic syndrome. Prompt identification of the exposure vector, rapid toxicological screening, and targeted chelation are fundamental to achieving sustained remission. Moving forward, studies should further elucidate the molecular mechanisms underlying mercury-induced podocyte injury and establish reliable strategies for identifying patients most likely to benefit from glucocorticoid therapy. Refining these diagnostic and therapeutic paradigms will mitigate the risks of empirical overtreatment and advance the individualized management of this cryptic condition.

## Data Availability

The original contributions presented in the study are included in the article/supplementary material, further inquiries can be directed to the corresponding author/s.

## References

[1] Wu C Liu D. Case report: nephrotic syndrome caused by mercury poisoning due to freckle-removing cream. Front Med. (2025) 12:1651441. doi: 10.3389/fmed.2025.1651441, 41282025 PMC12634324

[2] Ji H Chen Y Liu D Zhou T Tang Y. Diverse clinical manifestations and prognosis in a couple’s mercury poisoning caused by skin-lightening creams: two case reports and literature review. Front Med. (2024) 11:1511493. doi: 10.3389/fmed.2024.1511493, 39911669 PMC11794203

[3] Gao Z Wu N Du X Li H Mei X Song Y. Toxic nephropathy secondary to chronic mercury poisoning: clinical characteristics and outcomes. Kidney Int Rep. (2022) 7:1189–97. doi: 10.1016/j.ekir.2022.03.009, 35694560 PMC9174032

[4] Sun Y Long J Zhao J Peng X Qiu Z. Epidemiology, clinical presentation, treatment, and follow-up of chronic mercury poisoning in China: a retrospective analysis. BMC Pharmacol Toxicol. (2021) 22:25. doi: 10.1186/s40360-021-00493-y 33941274 PMC8091676

[5] Waack A Ranabothu M Vattipally V. Mercury poisoning from artisanal gold mining equipment. Radiol Case Rep. (2023) 18:607–9. doi: 10.1016/j.radcr.2022.10.099, 36465164 PMC9713274

[6] Barreto-Vazquez HD Rees M Yusuf S Harding SA Suen KP. The ayurvedic remedy rasa Karpura and a case of inorganic mercury toxicity. Pediatrics. (2025) 156:e2025071611. doi: 10.1542/peds.2025-071611, 41052789

[7] Edwards M Powell R. Mercury poisoning in a bodybuilder. Pract Neurol. (2024) 24:241–3. doi: 10.1136/pn-2023-003827, 38253381

[8] George J Sadiq E Moola I Maharaj S Mochan A. Informal gold miners with mercury toxicity: novel asymmetrical neurological presentations. S Afr Med J. (2023) 113:20. doi: 10.7196/SAMJ.2023.v113i12.1127, 38525630

[9] Liu C Huang Y Wei W Hu X Yang J Zhao Y. Mercury poisoning-associated membranous nephropathy and autoimmune encephalitis. BMC Nephrol. (2025) 26:148. doi: 10.1186/s12882-025-04082-7, 40128702 PMC11934491

[10] McNally MA Jenkinson H Narala S Rogge M. Palmoplantar eruption in a patient with mercury poisoning. Cutis. (2020) 106:265–7. doi: 10.12788/cutis.0113, 33465192

[11] Teschke R. Aluminum, arsenic, beryllium, cadmium, chromium, cobalt, copper, iron, lead, mercury, molybdenum, nickel, platinum, thallium, titanium, vanadium, and zinc: molecular aspects in experimental liver injury. Int J Mol Sci. (2022) 23:12213. doi: 10.3390/ijms232012213, 36293069 PMC9602583

[12] Teschke R Xuan TD. Heavy metals, halogenated hydrocarbons, phthalates, glyphosate, cordycepin, alcohol, drugs, and herbs, assessed for liver injury and mechanistic steps. Front Biosci (Landmark Ed). (2022) 27:314. doi: 10.31083/j.fbl2711314, 36472117

[13] Hogeland EW Somers TS Yip L Doyon S Redlich CA Orsey AD . Thrombocytopenia associated with elemental mercury poisoning in two siblings—Connecticut, July 2022. MMWR Morb Mortal Wkly Rep. (2023) 72:1027–31. doi: 10.15585/mmwr.mm7238a2, 37733629 PMC10519713

[14] Ansar A McNerney K Marshall BA Hebbard C Berg S Mullins M. Mercury toxicity mimicking pheochromocytoma. JCEM Case Rep. (2025) 3:luaf152. doi: 10.1210/jcemcr/luaf152, 40757054 PMC12315530

[15] Gao H Liu G He Y Chen J. Nephrotic syndrome of minimal change disease following exposure to mercury-containing skin lightening cream: a case report and literature review. Clin Nephrol. (2022) 98:107–12. doi: 10.5414/CN110751, 35603688

[16] Dong X Zhang XL Wan QJ. Mercury poisoning-induced minimal change disease with IgM deposition: a case report. Chin J Integr Med Nephrol. (2023) 24:454–6.

[17] Qin A Yu X Wang S Zhou F Zhao M. Unveiling the features of mercury-associated minimal change disease: comparison with primary minimal change disease. Kidney Dis. (2021) 7:156–66. doi: 10.1159/000510877, 33824871 PMC8010227

[18] Liu F Cui J Hu H Ren S Chen H Deng Y . Real-time tracking and early diagnosis of mercury (II) exposure via a novel NIR-based fluorescent probe based on thiahemicyanine dye. J Hazard Mater. (2025) 496:139462. doi: 10.1016/j.jhazmat.2025.139462, 40782779

[19] Zhou J Li J Xu X Long S Cui N Zhang Y . Imaging gastrointestinal damage due to acute mercury poisoning using a mitochondria-targeted dual near-infrared fluorescent probe. J Hazard Mater. (2024) 470:134269. doi: 10.1016/j.jhazmat.2024.134269, 38613952

[20] De Almeida EC Faria VD Cirinêu FD Santiago MGA Miotto B Vieira JCS . Metalloproteomic investigation of hg-binding proteins in renal tissue of rats exposed to mercury chloride. Int J Mol Sci. (2023) 25:164. doi: 10.3390/ijms25010164, 38203335 PMC10779082

[21] Pollard KM Cauvi DM Toomey CB Hultman P Kono DH. Mercury-induced inflammation and autoimmunity. Biochim Biophys Acta. (2019) 1863:129299. doi: 10.1016/j.bbagen.2019.02.001, 30742953 PMC6689266

[22] Balachandran A Jambugulam M George K Inturi S Padankatti S Alwan A . Clinical Spectrum of mercury poisoning in India: case-series from a poison control center. JAPI. (2023) 71:55–60. doi: 10.5005/japi-11001-0180, 37354482

[23] Bjørklund G Peana M Dadar M Chirumbolo S Aaseth J Martins N. Mercury-induced autoimmunity: drifting from micro to macro concerns on autoimmune disorders. Clin Immunol. (2020) 213:108352. doi: 10.1016/j.clim.2020.108352, 32032765

[24] Crespo-Lopez ME Barthelemy JL Lopes-Araújo A Santos-Sacramento L Leal-Nazaré CG Soares-Silva I . Revisiting genetic influence on mercury exposure and intoxication in humans: a scoping review. Toxics. (2023) 11:967. doi: 10.3390/toxics11120967, 38133368 PMC10747380

[25] Tavares LCP Caetano L d VN Ianhez M. Side effects of chronic systemic glucocorticoid therapy: what dermatologists should know. An Bras Dermatol. (2024) 99:259–68. doi: 10.1016/j.abd.2023.05.005, 38007314 PMC10943326

[26] Tang HL Mak YF Chu KH Lee W Fung SKS Chan TYK . Minimal change disease caused by exposure to mercury-containing skin lightening cream: a report of 4 cases. Clin Nephrol. (2013) 79:326–9. doi: 10.5414/cn107383, 23537684

[27] Niu HX Li SH Li HY Chen YH Liu WW Li PL . Clinicopathological features, diagnosis, and treatment of IgA nephropathy with minimal change disease related to exposure to mercury-containing cosmetics: a case report. Clin Nephrol. (2017) 87:196–201. doi: 10.5414/CN108967 28102816

[28] Zhang L Liu F Peng Y Sun L Chen C. Nephrotic syndrome of minimal change disease following exposure to mercury-containing skin-lightening cream. Ann Saudi Med. (2014) 34:257–61. doi: 10.5144/0256-4947.2014.257, 25266189 PMC6074594

[29] Wągrowska-Danilewicz M Danilewicz M Zbrog Z. Mercury-induced nephrotic syndrome: a case report and review of the literature. Pol J Pathol. (2014) 65:322–6. doi: 10.5114/pjp.2014.48194, 25693087

[30] Tang HL Chu KH Mak YF Lee W Cheuk A Yim KF . Minimal change disease following exposure to mercury-containing skin lightening cream. Hong Kong Med J. (2006) 12:316–8. 16912361

[31] Zhang SF Liu YQ Wu M Chen SJ Tang RN Pan YW . Whitening cosmetics-induced mercury poisoning-related renal injury: a case report. Chin J Nephrol. (2020) 36:397–9. doi: 10.3760/cma.j.cn441217-20191118-00075

